# Targeted RT study: results on early toxicity of targeted therapies and radiotherapy

**DOI:** 10.1186/s13014-024-02494-7

**Published:** 2024-08-29

**Authors:** Dinah Konnerth, Aurelie Gaasch, C. Benedikt Westphalen, Kathrin Heinrich, Maximilian Niyazi, Chukwuka Eze, Paul Rogowski, Sebastian Marschner, Annemarie Zinn, Claus Belka, Stefanie Corradini, Stephan Schönecker

**Affiliations:** 1 Department of Radiation Oncology, University Hospital, LMU Munich, Munich, Germany; 2 Partner Site Munich, German Cancer Consortium (DKTK), Munich, Germany; 3 Department of Medicine III, University Hospital, LMU Munich, Munich, Germany; 4 Comprehensive Cancer Center (CCC Munich LMU), University Hospital, LMU Munich, Munich, Germany; 5 Department of Radiation Oncology, University Hospital Tübingen, Tübingen, Germany; 6 Bavarian Cancer Research Center (BZKF), Munich, Germany

## Abstract

**Purpose/objective:**

Currently, there are few prospective data on the tolerability of combining targeted therapies (TT) with radiation therapy (RT). The objective of this prospective study was to assess the feasibility and toxicity of pairing RT with concurrent TT in cancer patients. The aim was to enhance the existing evidence base for the simultaneous administration of targeted substances together with radiotherapy.

**Methods:**

Prospective study enrollment was conducted at a single institution between March 1, 2020, and December 31, 2021, for all patients diagnosed with histologically confirmed cancer who underwent external beam radiotherapy in combination with targeted therapy. The study, known as the “targeted RT study,” was registered in the German Clinical Trials Register under DRKS00026193. Systematic documentation of the toxicity profiles of different targeted therapies was performed, and the assessment of acute toxicity followed the guidelines of the National Cancer Institute Common Terminology Criteria for Adverse Events Version v5.0.

**Results:**

A total of 334 patients underwent 683 radiation therapy series. During the course of RT, 51 different TT substances were concurrently administered. External beam radiotherapy was employed for various anatomical sites. The combination of RT and concurrent TT administration was generally well tolerated, with no instances of severe acute toxicity observed. The most commonly reported toxicity was fatigue, ranging from mild to moderate Common Terminology Criteria for Adverse Events (CTCAE) °I-°III. Other frequently observed toxicities included dermatitis, dyspnea, dysphagia, and dry cough. No toxicity greater than moderate severity was recorded at any point. In only 32 patients (4.7% of evaluated RT series), the concurrent substance administration was discontinued due to side effects. However, these side effects did not exceed mild severity according to CTCAE, suggesting that discontinuation was a precautionary measure. Only one patient receiving Imatinib treatment experienced a severe CTCAE °III side effect, leading to discontinuation of the concurrent substance due to the sudden occurrence of melaena during RT.

**Conclusion:**

In conclusion, the current study did not demonstrate a significant increase or additional toxicity when combining radiotherapy and concurrent targeted therapy. However, additional research is required to explore the specific toxicity profiles of the various substances that can be utilized in this context.

**Trial registration number:**

DRKS00026193. Date of registration 12/27/2022 (retrospectively registered).

## Introduction

Targeted therapies (TT) and radiation therapy (RT) are currently utilized in the treatment of various cancers, including common types like breast, lung, and colon cancer [1–4]. These therapies specifically target molecules or pathways involved in cancer cell growth and spread, inhibiting their progression. Targeted therapies can be used alone or in combination with chemotherapy and radiation therapy [5–7]. Examples of targeted therapies include monoclonal antibodies, kinase inhibitors, and signal transduction inhibitors [8].

Although targeted therapies are relatively new compared to other cancer treatment modalities, they have already demonstrated significant success. For example, locally advanced lung cancer was previously treated with radiation therapy and chemotherapy. However, the advent of targeted therapies has led to the examination of tumors using molecular genetic methods to identify potential targets for personalized treatment [9–11]. Nowadays, there is a wide range of specifically targetable structures, and research is progressing towards histology-agnostic therapies that focus on genomic alterations regardless of the primary tumor type [12].

Combining targeted therapies with radiation therapy has become increasingly prevalent in multimodal cancer treatment [13–15]. Examples of successful multimodal treatments include the Pacific Trial, where locally advanced lung carcinomas received chemoradiation followed by maintenance therapy with the monoclonal antibody durvalumab (anti-PD-L1), leading to improved overall survival [16]. Another notable study is the Checkmate 577, which extended the standard therapy for locally advanced esophageal cancer by incorporating maintenance therapy with the monoclonal antibody nivolumab (anti-PD-1), resulting in improved disease-free survival [17]. These findings have had a significant impact on clinical practice and were practice-changing.

However, both targeted therapies and radiation therapy can cause side effects such as fatigue, nausea, and skin irritation. When combined, these therapies may have additive or even synergistic side effects. For example, targeted therapies that inhibit specific proteins can lead to skin rashes or diarrhea [18, 19], while radiation therapy may cause skin irritation or diarrhea if the intestines are affected by radiation [20, 21]. Consequently, the combination of targeted therapies and radiation therapy and its impact on side effects are areas of active research in cancer treatment [22–24].

To date, there is limited prospective data on the tolerability of combining targeted therapies and radiation therapy. Therefore, the aim of this prospective study was to assess the feasibility and toxicity of combining concurrent targeted therapy with radiotherapy in cancer patients, with the goal of enhancing the evidence base for the simultaneous administration of targeted substances alongside radiation therapy. Although this approach has shown promise in many cases, further data on its effectiveness and safety are still required.

## Methods

Data for this prospective study were collected at LMU University Hospital from March 1, 2020, to December 31, 2021. The targeted RT study, was registered in the German Clinical Trials Register under DRKS00026193. The study enrolled all patients with histologically confirmed cancer who underwent both radiotherapy and concurrent targeted therapy. Informed consent was obtained from all participants. Data were extracted from medical records. The toxicity profiles of different targeted therapies were systematically documented at the time of first irradiation as baseline, the final irradiation, and during follow-up visits at our institution. The assessment of toxicity followed the guidelines of the National Cancer Institute Common Terminology Criteria for Adverse Events Version v5.0 [25].

Comprehensive demographic data and treatment characteristics were recorded, encompassing age, sex, Karnofsky Performance Status Scale, diagnosis according to International Statistical Classification of Diseases and Related Health Problems 10 (ICD-10), survival status, follow-up information, and timing of irradiation. Additionally, entity-specific toxicities that may have been present at baseline were documented to facilitate a thorough comparison between the beginning and end of the RT treatment. The specific anatomic region subjected to irradiation was recorded along with the determination of the target volume size (planning target volume, PTV). Furthermore, the fraction dose, total dose, and number of fractions administered in each RT series were evaluated, considering the employed techniques such as volumetric intensity-modulated arc therapy (VMAT), 3-dimensional radiation therapy (3D-RT), stereotactic body radiation therapy (SBRT), dynamic multileaf collimator radiation therapy (dMLC-RT), intensity-modulated radiation therapy (IMRT), brachytherapy, or electrons, as well as the intent of irradiation (definitive, adjuvant, neo-adjuvant, local-ablative, palliative, consolidative, prophylactic). The occurrence of previous in-field RT was also documented, as this information is essential for assessing toxicities during the course of irradiation.

The dataset also encompassed comprehensive information regarding systemic therapies. The primary focus was on targeted substances, specifying the substance used and the number of targeted substances administered concurrently to RT. Additionally, information was collected on whether chemotherapy was administered simultaneously or in close temporal relation to irradiation, along with the specific substances involved. For targeted substances, the timing of their application was documented, as well as whether they were paused during radiation. If paused, the duration of the break (< or ≥ than one week) was noted to ensure satisfactory assessment of toxicities relative to the time interval before irradiation. A break shorter than one week before the start of irradiation treatment was considered simultaneous application. For patients receiving cerebral irradiation, the use of dexamethasone during irradiation was also recorded.

Toxicities were evaluated taking into account the specific tumor entity and the anatomical region undergoing radiotherapy, covering a wide range of 54 different items of toxicities across all tumor types. Whenever feasible, an attempt was made to discern whether a particular toxicity was associated with the irradiation or could be attributed to the targeted therapy being administered. The ASENA database [26] was employed as the data collection tool, enabling the generation and customization of data forms tailored to the specific requirements of the study. Data extraction from ASENA was conducted, and the extracted data were further analyzed using Excel.

## Results

A total of 334 patients underwent 683 radiation therapy series and the administration of 51 different targeted therapy substances. A comprehensive overview of the administered TT substances is provided in Table 1, which includes various classes such as protein kinase inhibitors (including serine/threonine kinase inhibitors, tyrosine kinase inhibitors, and multikinase inhibitors), selective estrogen receptor degraders, CDK4/6 inhibitors, PARP inhibitors, mTOR inhibitors, proteasome inhibitors, Bcl-2 inhibitors, and bispecific gp100 peptide-HLA-directed CD3 T cell engagers. Among the 683 RT series, 314 (46%) did not have targeted therapy paused or had a pause duration of less than one week before initiating irradiation treatment. Moreover, in 106 RT series (16%) chemotherapy was applicated simultaneously to TT.

**Table 1:** Targeted therapy substances administered

TT (In alphabetical order)	Type of TT (Target in parentheses)
Abemaciclib	CDK4/6 inhibitor
Adalimumab	monoclonal antibody (TNF alpha)
Afatinib	TKI (EGFR)
Alectinib	TKI (ALK/RET)
Atezolizumab	monoclonal antibody (PD-L1)
Axitinib	TKI (VEGFR)
Bevacizumab	monoclonal antibody (VEGF-A)
Binimetinib	protein kinase inhibitor (MEK)
Bortezomib	proteasome inhibitor
Cabozantinib	multikinase inhibitor
Cetuximab	monoclonal antibody (EGFR)
Crizotinib	TKI (ALK/ROS1)
Dabrafenib	protein kinase inhibitor (B-Raf)
Daratumumab	monoclonal antibody (CD-38)
Denosumab	monoclonal antibody (RANKL)
Durvalumab	monoclonal antibody (PD-L1)
Encorafenib	protein kinase inhibitor (B-Raf)
Erlotinib	TKI (EGFR)
Everolimus	protein kinase inhibitor (mTOR)
Fulvestrant	SERD
Gefitinib	TKI (EGFR)
Ibrutinib	TKI (BTK)
Imatinib	TKI (multiple tyrosine kinases)
Ipilimumab	monoclonal antibody (CTLA-4)
Ixazomib	proteasome inhibitor
Lenalidomide	immunmodulatory drug
Lenvatinib	multi kinase inhibitor
Lorlatinib	TKI (ALK/ROS1)
Nintedanib	TKI (VEGFR, FGF, PDGF)
Niraparib	PARP inhibitor
Nivolumab	monoclonal antibody (PD-1)
Obinutuzumab	monoclonal antibody (CD-20)
Olaparib	PARP inhibitor
Osimertinib	TKI (EGFR)
Palbociclib	CDK4/6 inhibitor
Panitumumab	monoclonal antibody (EGFR)
Pembrolizumab	monoclonal antibody (PD-1)
Pertuzumab	monoclonal antibody (Her2)
Polatuzumab	monoclonal antibody (CD79B)
Ponatinib	TKI (multiple tyrosine kinases)
Ramucirumab	monoclonal antibody (VEGFR2)
Ribociclib	CDK4/6 inhibitor
Rituximab	monoclonal antibody (CD 20)
Sorafenib	multi kinase inhibitor
Sunitinib	TKI (multiple tyrosine kinases)
Tafasitamab	monoclonal antibody (CD 19)
Tebentafusp	bispecific gp100 peptide-HLA-directed CD3 T cell engager
Tivozanib	TKI (VEGFR)
Trametinib	protein kinase inhibitor (MEK)
Trastuzumab	monoclonal antibody (Her2)
Venetoclax	Bcl-2 inhibitor
Total of 51 different substances	

The RT treatments were performed across various anatomic sites, including the head and neck region (6%), central nervous system (25%), thoracic (32%), breast (8%), abdominal/pelvic region (24%), and extremities (4%). Additionally, in 1% RT was administered to overlapping regions i.e. the RT fields extended over more than one anatomical region (e.g. in the case of RT of extensive bone metastases by way of example RT from T10 to L2 includes the thoracic and the abdominal regions). Treatment intent varied, with the most common indications being local-ablative (37%) and palliative (37%). However, adjuvant, definitive, consolidating, prophylactic, and neoadjuvant treatments were also performed. A prior irradiation was present in 169 out of the total 334 patients, accounting for 51% of the cases. Some of these series involved field overlaps in the low dose range and 8% of irradiation series even involved true re-irradiation or overlap in the high dose range.

**Fig. 1:**
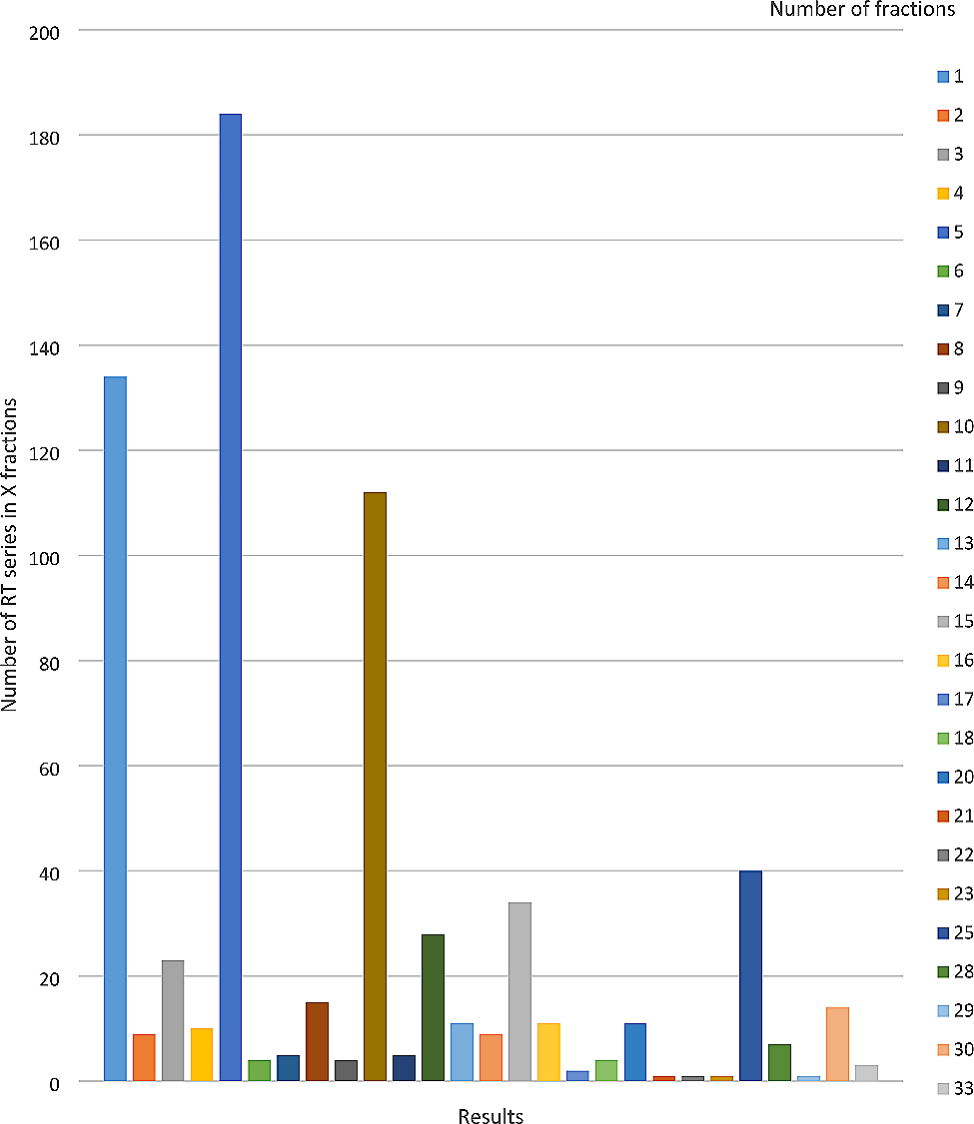
Number of fractions and amount of RT series with corresponding fractionation

There were varying numbers of fractionation regimens administered in the irradiation series. A detailed overview is given in Fig. 1. The most frequent were single fraction (134/683), 5 fractions (184/683) and 10 fraction regimens (112/683). In total, 5998 fractions were applied. The single dose varied considerably depending on treatment intention, localization, and tumor entity and ranged from 2 Gy to local ablative single fraction irradiations with a single dose of 28 Gy.

In 32 RT series (4.7%) TT was discontinued due to side effects not tolerated by the patient or on the advice of the treating physician. Table 2 summarizes the patient characteristics and treatment details.

**Table 2:** Patient characteristics and treatment details of the entire cohort (334 patients and 683 radiation therapy series)

Patient characteristics and treatment details	Characteristic	*n* (%)
**Gender**	male	= 149 (44.6%)
	female	= 185 (55.4%)
**Treatment intent**	palliative	= 252 (37%)
	local-ablative	= 253 (37%)
	adjuvant	= 104 (15%)
	definitive	= 49 (7%)
	consolidating	= 17 (3%)
	neoadjuvant	= 6 (1%)
	prophylactic	= 2 (0%)
**Irradiated region**	thoracic	= 219 (32%)
	CNS (brain)	= 169 (25%)
	abdominal/pelvic	= 162 (24%)
	breast	= 55 (8%)
	head & neck	= 44 (6%)
	extremities	= 27 (4%)
	overlapping regions	= 7 (1%)
**Number of RT fractions**	see Fig. 1	
**Re-RT**	yes	= 55 (8%)
	no	= 628 (92%)
**Type of TT**	monoclonal antibody	= 511 (75%)
	protein kinase inhibitor	= 100 (15%)
	CDK4/6 inhibitor	= 35 (5%)
	proteasome inhibitor	= 11 (2%)
	PARP inhibitor	= 8 (1%)
	immunomodulatory drug	= 8 (1%)
	SERD	= 6 (1%)
	Bcl-2 inhibitor	= 3 (0%)
	bispecific gp100 peptide-HLA-directed CD3 T cell engager	= 1 (0%)
**TT paused**	yes	= 369 (54%)
	no	= 314 (46%)
**Simultaneous chemotherapy**	yes	= 106 (16%)
	no	= 577 (84%)

The following table (Table 3) provides an overview of the recorded toxicities at the three time points “baseline“, “end of irradiation“ and “follow-up“. Owing to the limited number of events recorded, a shift to a descriptive evaluation approach was warranted, underlining the challenge posed by low event rates in achieving statistical significance for discerning differences in side effect profiles.

The most frequently observed side effect across all time points was fatigue. It also increased steadily at all evaluation time points. Baseline observations also revealed skin side effects, attributed to patients undergoing repeated series of irradiation within the same field. At the end of irradiation of course, there was an upward trend in side effects. A trend was identified by analyzing the data for a consistent increase in the frequency of side effects over a specified period of time. This involved looking at the side effects data points across different time intervals and observing a general pattern (e.g. from baseline to end of irradiation, see Table 3). In particular, the side effects concerning the skin, as well as the ear, nose and throat (ENT) side effects showed an upward trend. The first follow-up was performed after an average of about 3 months. The timing of further follow-ups depended on the tumor entity. Some follow-up visits continued to take place every three months, while subsequent follow-up visits varied based on tumor entity, with some maintaining a quarterly schedule, while others deferred the second radiotherapy follow-up for a year. The mean of all recorded follow-up visits was 6.3 months (SD = 4.2 months) after the end of radiotherapy. To account for varying follow-up intervals, the subsequent table provides an inclusive view of toxicities across all follow-up periods, assessing regression, exacerbation, or the emergence of new toxicities post-radiotherapy.

The follow-up again showed a downward trend in acute skin as well as in dysphagia side effects, but an upward trend in late side effects such as hyperpigmentation. A downward trend was also observed in ENT side effects. When addressing side effects after central nervous system (CNS) irradiation, a subtle inclination towards increased adverse events was apparent during the follow-up.

The additional surveyed toxicities are excluded from this report since they were observed even in CTCAE °I in less than 5 radiation series and exhibited minimal or negligible shifts from baseline to end of irradiation and to follow-up. Overall, no fourth- or fifth degree toxicity was observed.

**Table 3:** Overall toxicities and corresponding increasing or decreasing trends recorded during different time points of the treatment and during follow-up

Toxicity	CTCAE degree	Baseline frequency (Number of patients)	End of irradiation frequency (Number of patients)	Trend	Follow-up frequency (Number of patients)	Trend
Alopecia	°I	4	10	↑	14	↑
	°II	1	2	↑	15	↑
	°III	0	0	→	2	↑
Impaired Concentration	°I	9	15	↑	16	↑
	°II	2	1	↓	3	↑
	°III	0	0	→	0	→
Impaired Memory	°I	10	7	↓	15	↑
	°II	1	1	→	2	↑
	°III	0	0	→	0	→
Vertigo	°I	11	8	↓	17	↑
	°II	0	0	→	1	↑
	°III	0	0	→	0	→
Fatigue	°I	65	75	↑	79	↑
	°II	11	16	↑	11	↓
	°III	1	1	→	0	↓
Dysphagia	°I	16	20	↑	12	↓
	°II	1	4	↑	1	↓
	°III	1	3	↑	2	↓
Mucositis	°I	0	5	↑	0	↓
	°II	0	1	↑	1	→
	°III	0	0	→	0	→
Xerostomia	°I	1	5	↑	7	↑
	°II	0	1	↑	0	↓
	°III	0	0	→	0	→
Hyperpigmentation	°I	5	7	↑	9	↑
	°II	0	1	↑	0	↓
	°III	0	0	→	0	→
Radiogenic Dermatitis	°I	11	34	↑	9	↓
	°II	1	11	↑	0	↓
	°III	0	2	↑	0	↓
Fibrosis	°I	5	7	↑	14	↑
	°II	0	0	→	1	↑
	°III	0	0	→	0	→
Breast edema	°I	0	4	↑	4	→
	°II	0	0	→	0	→
	°III	0	0	→	0	→
Dyspnea	°I	32	22	↓	35	↑
	°II	1	0	↓	7	↑
	°III	1	1	→	0	↓
Dry Cough	°I	23	24	↑	36	↑
	°II	3	1	↓	1	→
	°III	0	0	→	0	→
Nausea	°I	1	4	↑	2	↓
	°II	0	0	→	0	→
	°III	0	0	→	0	→
Pain	°I	2	6	↑	6	→
	°II	2	2	→	0	↓
	°III	0	0	→	0	→
Dysuria	°I	1	1	→	1	→
	°II	0	0	→	0	→
	°III	0	0	→	0	→
Urinary Incontinence	°I	1	1	→	1	→
	°II	0	0	→	0	→
	°III	0	0	→	0	→
Nycturia	°I	3	3	→	5	↑
	°II	0	1	↑	0	↓
	°III	0	0	→	0	→

## Discussion

Overall, our dataset shows a favorable toxicity profile, across all irradiated regions and across all targeted therapies. There was no type of TT, in which particularly pronounced side effects could be observed. It is noteworthy that despite half of the patients having received more than one irradiation series, which inherently complicates toxicity assessment, and considering the elevated risk associated with re-irradiation, toxicity beyond grade 3 has remained conspicuously absent. While thorax irradiation accounted for the highest overall toxicity rates, these levels remain within an acceptable range, and are most likely reversible in the longer follow-up and/or with treatment. Expectedly, fatigue was the predominant toxicity, followed by dyspnea and dry cough.

Smets et al.‘s investigation on the relationship between radiotherapy and fatigue revealed that fatigue scores post-treatment were slightly elevated compared to pretreatment levels, and 46% of their study participants reported fatigue after irradiation treatment [27]. Their subsequent follow-up analysis indicated that 39% still experienced relevant fatigue symptoms nine months post-treatment [28] Therefore, one might conclude, that fatigue is in general a very common symptom and is not enhanced due to simultaneous TT especially since fatigue values in our study population were much lower than that with around 13%.

Similarly the second and third most reported toxicities, dyspnea and dry cough, are also side effects that are related to radiotherapy - even without additional systemic therapy [29]. A study by Tsoutsou et al. showed that thorough assessment indicates that 50–90% of patients receiving thoracic irradiation develop irregularities that are evident both radiographically and in pulmonary function tests [30]. Remarkably, no excessive accumulation of these side effects that would make themselves felt via dyspnea and dry cough, was detected within our cohort, aligning with empirical values from our institution (e.g. clinical records, follow-up data and patient surveys). Those side effects commonly follow a distinctive trajectory, often intensifying subsequent to radiotherapy. The emergence of pneumonitis aligns with this pattern, becoming evident around one to three months after the completion of radiotherapy [31]. This temporal connection is reflected in an increase in dyspnea and dry cough in our follow-up examinations as well.

Combining targeted therapies with radiation therapy can be an effective way to treat cancer. Nevertheless, informed decision-making necessitates a balanced consideration of potential advantages and disadvantages. A primary asset of combining radiation therapy with targeted medication is the precise targeting of cancer cell growth and proliferation pathways, conferring greater efficacy compared to conventional chemotherapy [5].

This amalgamation holds promise for improved outcomes by targeting specific molecular pathways crucial for cancer cell survival, while radiation therapy imparts cytotoxic damage or death to cancer cells through ionizing radiation [32–34]. Moreover, targeted therapies can sensitize tumors to radiation, augmenting radiotherapy’s potency [35, 36].

Targeted therapies may be less toxic and may have fewer side effects than traditional chemotherapy, making them a good option for patients who are unable to tolerate potential side effects of chemotherapy [37]. Additionally, the prospect of reduced radiation doses through combination therapy has the potential to mitigate side effects and long-term complications [38]. In their consensus recommendations, the EORTC–ESTRO OligoCare consortium identified a clinically significant increase in SBRT-induced thoracic and abdominal toxicity when SBRT was combined with certain monoclonal antibodies or kinase inhibitors, including ipilimumab, nivolumab, bevacizumab, and sorafenib. Conversely, combining EGFR, ALK, and mTOR inhibitors with metastases-directed SBRT did not show increased toxicity rates. Regarding the same-day administration of targeted therapies and SBRT, consensus was reached for only some substances. Similarly, consensus on the duration of interruption before and after SBRT was also achieved for only some targeted therapies. The authors acknowledge that the limited data available made it challenging to reach a consensus on the simultaneous application for all substances and the appropriate duration of treatment interruption [39].

The integration of chemotherapy and radiotherapy has already been established as a well-acknowledged therapeutic modality within the landscape of cancer treatment. In the context of this study, a subset of our patient cohort underwent concurrent administration of chemotherapy and radiotherapy (16%). It is noteworthy that this proportion might initially appear moderate; however, this observation can be attributed to the fact that this study included patients who were concurrently undergoing targeted therapy at the time of radiotherapeutic intervention. As of now, targeted therapeutic approaches typically assume a secondary role in the therapeutic hierarchy, reserved for advanced cancer stages after the depletion of conventional treatment modalities, which predominantly encompass chemotherapy. Along with this, in this study population, the percentage of palliative intended radiotherapy (also rather a treatment for advanced stages) is quite high (37%).

Decisions regarding the continuity or interruption of targeted therapy during the course of radiotherapy were contingent upon multifaceted considerations. Initially, our determinations were underpinned by the guidance furnished by the Radiation Safety Commission (Strahlenschutzkommission [40]). Nevertheless, it is imperative to acknowledge that the aforementioned regulatory source does not comprehensively encompass the entire gamut of current targeted agents. Consequently, a broader spectrum of determinants, encompassing the patient’s overall medical condition and the tolerability of the targeted therapeutic regimen leading up to the radiotherapeutic intervention, were influential in the decision-making process.

Furthermore, it merits attention that a considerable proportion of targeted therapeutic agents are administered in temporal intervals spanning 3 to 4 weeks. Even in instances where the targeted regimen was not deliberately halted, meticulous consideration was exercised to avert any precise overlap between individual radiotherapy sessions and the administration of the therapeutic agent. This practice, however, encountered exceptions, notably in the context of orally administered agents such as CDK4/6 inhibitors that necessitate daily consumption, whereupon radiation therapy and targeted therapy coincided exactly.

Instances wherein the targeted therapeutic regimen required interruption were infrequent (4.7%). Such decisions predominantly emanated from a precautionary standpoint, owing to the prevailing dearth of robust empirical evidence substantiating the concurrent utilization of these therapeutic modalities. The rationale behind discontinuation encompassed a spectrum of factors, inclusive of a general decline in overall health status, fatigue or cutaneous reactions. However, it remains uncertain, whether these occurrences should be solely attributed to the combined therapeutic approach. Of particular significance is a solitary case wherein a patient afflicted with a gastrointestinal stromal tumor (GIST) was treated with imatinib—a protein kinase inhibitor—and concurrently received palliative radiotherapy at our institution. In this patient, a notable adverse effect, specifically melaena, manifested, necessitating the suspension of the targeted therapeutic regimen. Nevertheless, it is reasonable to infer that this phenomenon is unlikely to be causally related to the parallel administration of palliative radiotherapy [41].

In spite of diligent efforts invested in conducting this study, it is essential to acknowledge the presence of limitations that warrant thoughtful consideration. One of the main reasons was the COVID pandemic. Due to the COVID restrictions imposed in Germany, some follow-ups during the period in which this study was conducted were done by telephone if possible. Thus, it is of course significantly more difficult to assess the side effects adequately, one has to rely to a large extent on the assessment of the patient. It might be the case that patients do not report side effects or do not recognize a side effect as such [42]. There may also be disincentives for reporting side effects, such as fear of treatment discontinuation possibly leading to a worse oncological outcome. In addition, while the evaluation of toxicities by means of CTCAE is a very good tool which tries to give objective criteria to the evaluator, there is always a risk for interobserver variability. In our facility, patients in this study were seen and assessed by many different physicians, so these differences in assessment may play a role after all [43–45]. Other limitations could be the unawareness of the complete spectrum of potential side effects linked to specific therapies. This can include a lack of reporting from healthcare providers, who sometimes might not be aware of the full range of potential side effects for a particular therapy and as a result may not report all of the side effects that they observe in their patients.

Moreover, inpatient care is available in our facility for cases where outpatient radiation is unfeasible due to deteriorated health. Timely inpatient admission, already at the onset of radiation induced side effects and treatment by specialized medical and nursing staff, effectively prevents the development of severe side effects and contributes to our favorable results. These limitations might underscore the complexity of the clinical landscape and the necessity for cautious interpretation of our findings.

Despite potential data limitations, our current findings affirm the safe administration of targeted therapies combined with radiation therapy. Although every medical treatment bears potential for side effects, our study witnessed scarce high-grade side effects in patients undergoing this combination, suggesting a low risk of severe complications. It is essential to note that effective side effect management in Germany contributes to this outcome, with early intervention preventing the escalation of toxicities. These combined therapies exhibit promising results across various cancer types, and the continual emergence of novel targeted therapies further underscores the importance of investigating combination therapy safety. Utilized in conjunction with radiation therapy, targeted therapies hold the potential to amplify treatment effectiveness while potentially reducing radiation dosage.

It is important to note that our data may not be comprehensive and there may be other factors that could affect the safety of this treatment combination. Further research is needed to fully understand the long-term effects of targeted therapies in combination with radiation therapy. However, our present data supports the safe application of this combined modality in clinical practice. Future research should persist in exploring the long-term tolerability of radiation and targeted therapy combinations, including a focus on potential changes in laboratory values as well as in exploring the efficacy of this combination treatment.

In summary, the combination of targeted therapies and radiation therapy emerges as a safe approach in cancer treatment. However, each patient’s individualized treatment plan must meticulously weigh the potential benefits and drawbacks. Collaborative interdisciplinary decision-making is paramount in optimizing patient outcomes.

## Data Availability

No datasets were generated or analysed during the current study.
